# Impact of the TLR4 agonist BECC438 on a novel vaccine formulation against *Shigella* spp.

**DOI:** 10.3389/fimmu.2023.1194912

**Published:** 2023-09-06

**Authors:** Ti Lu, Sayan Das, Debaki R. Howlader, Akshay Jain, Gang Hu, Zackary K. Dietz, Qi Zheng, Siva Sai Kumar Ratnakaram, Sean K. Whittier, David J. Varisco, Robert K. Ernst, William D. Picking, Wendy L. Picking

**Affiliations:** ^1^ Department of Pharmaceutical Chemistry, University of Kansas, Lawrence, KS, United States; ^2^ Department of Veterinary Pathobiology, University of Missouri, Columbia, MO, United States; ^3^ Department of Microbial Pathogenesis, School of Dentistry, University of Maryland, Baltimore, MD, United States

**Keywords:** *Shigella*, T3SS, IL-17, IFN-gamma, vaccine

## Abstract

Shigellosis (bacillary dysentery) is a severe gastrointestinal infection with a global incidence of 90 million cases annually. Despite the severity of this disease, there is currently no licensed vaccine against shigellosis. *Shigella*’s primary virulence factor is its type III secretion system (T3SS), which is a specialized nanomachine used to manipulate host cells. A fusion of T3SS injectisome needle tip protein IpaD and translocator protein IpaB, termed DBF, when admixed with the mucosal adjuvant double-mutant labile toxin (dmLT) from enterotoxigenic *E. coli* was protective using a murine pulmonary model. To facilitate the production of this platform, a recombinant protein that consisted of LTA-1, the active moiety of dmLT, and DBF were genetically fused, resulting in L-DBF, which showed improved protection against *Shigella* challenge. To extrapolate this protection from mice to humans, we modified the formulation to provide for a multivalent presentation with the addition of an adjuvant approved for use in human vaccines. Here, we show that L-DBF formulated (admix) with a newly developed TLR4 agonist called BECC438 (a detoxified lipid A analog identified as Bacterial Enzymatic Combinatorial Chemistry candidate #438), formulated as an oil-in-water emulsion, has a very high protective efficacy at low antigen doses against lethal *Shigella* challenge in our mouse model. Optimal protection was observed when this formulation was introduced at a mucosal site (intranasally). When the formulation was then evaluated for the immune response it elicits, protection appeared to correlate with high IFN-γ and IL-17 secretion from mucosal site lymphocytes.

## Introduction

*Shigella* causes bloody diarrhea (dysentery), which, with 90 million cases globally each year (1), results in approximately 164,000 deaths (2). Children are especially vulnerable, with repeated episodes causing developmental and cognitive impairment (3–5). *Shigella* is also an important cause of diarrhea among travelers and military personnel who visit low- and middle-income countries (2). *Shigella* spp. are classified into *S. dysenteriae* (Group A), *S. flexneri* (Group B), *S. boydii* (Group C), and *S. sonnei* (Group D), which can be further divided into more than 50 serotypes based on the O-antigen component of lipopolysaccharide (2). *S. flexneri*, which includes 19 serotypes, is the primary species responsible for endemic shigellosis in developing countries (2). In contrast*, S. sonnei*, which comprises a single serotype, is predominant in more developed countries (6). Recent studies have shown that *S. sonnei* infections are increasing and are replacing endemic *S. flexneri* infections in some areas, which calls for the strategic development of a serotype-independent vaccine to reduce the worldwide infection burden (6).

Antibiotics, such as fluoroquinolones, β-lactams, and cephalosporins, have proven to be effective in reducing the risk of serious complications and death from shigellosis (7); however, increased resistance in developing countries has become a major concern in the treatment of shigellosis (8). Therefore, developing an effective and safe vaccine against shigellosis will be an important strategy for reducing mortality and limiting antibiotic resistance. At present, there is no licensed vaccine against *Shigella*. There are some vaccine candidates, such as killed cell vaccines and live attenuated vaccines, which are currently being used in clinical trials; however, low immunogenicity, lack of cross-protection, strict storage conditions, and the potential risks of contamination limit their use in developing countries (9).

To solve these issues, subunit vaccines, especially those comprised of the proteins from the type three secretion system (T3SS) (see **Supplementary Table S1** for a table of the acronyms used here), have been widely researched (10). T3SS is an important virulence factor used by *Shigella* to inject virulence effectors into host cells to facilitate cellular entry and to escape the host immune response (11). We have previously demonstrated that invasion plasmid antigen D (IpaD) resides at the tip of the *Shigella* T3SS injectisome needle and is required for the control of type III secretion (12). We have also shown that the translocator protein IpaB associates with IpaD at the tip of the needle and makes initial contact with host cells (13). IpaD and IpaB are highly conserved among the shigellae, which makes them potential targets for the development of a serotype-independent subunit vaccine against *Shigella* spp. Studies in our laboratory have established that these two proteins administered with the mucosal adjuvant dmLT (double mutant heat-labile enterotoxin from ETEC) can elicit cross-protection against *S. flexneri* and *S. sonnei* when delivered intranasally (IN) (14). To reduce production costs, we produced a genetic fusion of IpaB and IpaD, termed DBF (15). DBF administered with dmLT elicited a comparable immune response with protection similar to that stimulated by the mixture of IpaB and IpaD (15). Most importantly, DBF with dmLT administered IN protected mice from *S. flexneri*, *S. sonnei*, and *S. dysenteriae* homologous and heterologous challenges (15).

Th17 responses, such as those elicited by dmLT, are believed to be important for protection against *Shigella* spp (16).. Unfortunately, studies have shown that dmLT, when delivered IN, can cause Bell’s palsy in humans (17, 18). Since the LTA1 portion of the A subunit is responsible for generating the Th17 response (19), we genetically fused LTA1 to DBF to create a monomeric adjuvant-antigen conjugate L-DBF (20). The absence of the LT B subunit abrogates toxins binding to ganglioside GM_1_ on neuronal cells in the nasal passage and eliminates the risk of Bell’s palsy (19). When delivered IN, L-DBF protects mice against homologous and heterologous *Shigella* spp. challenges. This protection is associated with significant Th1 and Th17 responses (20).

While L-DBF has been shown to successfully protect mice against lethal *Shigella* challenge, monomeric antigens often fail once they are introduced into human trials (21, 22). Studies have shown a better response is elicited in humans when the antigen is presented as a multimer in the context of a nanoparticle (21, 22). There are nanoparticle formulations currently being tested for use in intramuscular and intranasal vaccines (23–25). The most well-known multimerization method is the use of aluminum salts such as Alhydrogel; however, aluminum salts tend to skew the resulting immunity to a Th2 response, which is more aligned with the humoral response and not the balanced responses often required for clearing mucosal pathogens (26).

In this study, we examine the efficacy of DBF when formulated with the Bacterial Enzymatic Combinatorial Chemistry candidate 438 (hereafter referred to as BECC438), a novel TLR-4 agonist that is a biosimilar of monophosphoryl lipid A (MPL), which is approved for use in some human vaccines (26). BECC438 is a bis-phosphorylated hexa-acylated lipid A prepared from specifically engineered strains of *Yersinia pestis*. These studies were followed by an exploration of L-DBF formulated with BECC438 in an oil-in-water emulsion containing squalene, which has been shown to promote protection against influenza in an older population (27). In addition to fusion with LTA1, the use of BECC438 further promotes a balanced Th1-Th2 immune response and increases protection elicited by low doses of L-DBF.

## Methods

### Materials

pACYCDuet-1 plasmid, ligation mix, and competent *E. coli* were from EMD Millipore (Billerica, MA). Restriction endonucleases were from New England Biolabs (Ipswich, MA). Chromatography columns were from GE Healthcare (Piscataway, NJ). All other reagents were from Sigma or Fisher Scientific and were chemical grade or higher. dmLT was a gift from J. Clements and E. Norton, Tulane School of Medicine, New Orleans, LA. *S. flexneri 2a* 2457T was a gift from A.T. Maurelli, University of Florida, Gainesville, Fl. Squalene was from Echelon Biosciences (Salt Lake City, UT).

### Protein production

IpaD, IpaB, DBF, and L-DBF were made as previously described (14, 15, 20). Briefly, we utilized IpaD and IpaB proteins from *S. flexneri* 2a (strain 2457T). These proteins were chosen based on their high conservation across different serotypes of *Shigella* (IpaB: 98.9%; IpaD: 96%), as well as their known immunogenicity and involvement in the virulence of *Shigella* bacteria. The LTA (heat-labile toxin A1 subunit) used in our vaccine formulations was obtained from enterotoxigenic *E. coli* (ETEC). The final L-DBF preparation was dialyzed into PBS with 0.05% lauryl-dimethylamine oxide (LDAO) and stored at -80°C. LPS levels were determined using a NexGen PTS with EndoSafe cartridges (Charles River Laboratories, Wilmington, MA). All proteins had LPS levels <5 Endotoxin units/mg.

### Preparation of vaccine formulations

Squalene (8% w/v) and polysorbate 80 (2% w/v weight) were mixed to achieve a homogenous oil phase. Polysorbate 80 was used as an emulsifying agent to stabilize the emulsion. Using a Silverson L5M-A standard high-speed mixer, 40 mM histidine (pH 6) and 20% sucrose were added to the oil phase and mixed at 7500 RPM, followed by six passes in a Microfluidics 110P microfluidizer at 20,000 psi to generate a milky emulsion of 4XME (MedImmune Emulsion) (28). A similar method was used to make 4X NE (a new squalene-based emulsion formulated in our laboratory using a different aqueous phase) using 40 mM MOPS/20 mM Na_2_HPO_4_ (pH 7.6). To make the DBF with ME or NE, the protein was added to the emulsion to a final concentration of 0.67 mg/ml, vortexed, and allowed to incubate overnight at 4°C. To the emulsions containing BECC438, BECC438 (2 mg/ml) was prepared in 0.5% triethylamine by vortexing, followed by sonicating for 30 min in a 60°C water bath sonicator until the BECC438 was completely dissolved. The pH of BECC438 solution was adjusted to 7.2 with 1 M HCl. To make the BECC438 with ME or NE formulation, the BECC438 was mixed with ME or NE and the sample was vortexed for 2 min followed by overnight incubation at 4°C. The next day, DBF or L-BDF was mixed with the appropriate base formulation at a volumetric ratio of 1:1 to achieve the desired final antigen concentration.

### Preparation of DBF BECC438/Chi-C48/80 formulation

To make chitosan nanoparticles, 1 gm of chitosan was added to 10 mL of a 1 M NaOH solution and stirred for 3 h at 50°C (29). The chitosan solution was then filtered through a 0.45 µm membrane and the solid fraction was washed with 20 mL of MilliQ water. The recovered chitosan was resuspended in 200 mL of 1% (v/v) acetic acid solution and stirred for 1 h. The solution was filtered through a 0.45 µm membrane and 1 M NaOH was added to adjust the pH to 8.0, resulting in purified chitosan. Purified chitosan was vacuum dried for 24 hours at 40°C. The mast cell activating agent compound 48/80 (C48/80) was then loaded on the chitosan nanoparticles (Chi), adding dropwise 3 ml of an alkaline solution (5 mM NaOH) containing C48/80 and Na_2_SO_4_ (0.3 mg/mL and 2.03 mg/mL, respectively) to 3 mL of a chitosan solution (1 mg/mL in acetic acid 0.1%) with high-speed vortexing. The Chi was formed using magnetic stirring for an additional 1 h (30). Chi was then collected by centrifugation at 4500Xg for 30 min and the pellet was resuspended in MOPS buffer (20 mM, pH 7). The DBF in PBS was exchanged into MOPS buffer (20 mM, pH 7) using an Amicon Ultra-4 centrifugal filter. To make DBF BECC438/Chi-C48/80, the nanoparticles were mixed with BECC438 by vortexing and incubating for 10 min. DBF was then added, mixed by vortexing, and incubated for 2 h at 4°C.

### Mouse immunization and sample collection

The mouse animal protocols were reviewed and approved by the University of Kansas Institutional Animal Care and Use Committee Practices (protocol AUS 222-01). Female 6-8 week-old BALB/c mice were used in this study (n=10 or 14/group). The negative control group was vaccinated with PBS (30 µl), either intranasally (IN), intramuscularly (IM), or intradermally (ID) depending upon the route of the experimental groups. The positive control group was vaccinated IN with 20 µg DBF + 2.5 µg dmLT or 25 µg L-DBF. For the IM trials, the indicated formulations were prepared in 30 µl volume and delivered to the inner thigh with a 1/2 cc LO-DOSE U-100 Insulin Syringe with a 28G ½” needle. For the IN trials, the indicated formulations were prepared in 30 µl volumes and delivered with a pipette tip to the nares. Mice were immunized on Days 0, 14, and 28. For the ID trial, 100, 250, and 500 ng DBF with 5 µg BECC438 were diluted to 50 µl per mouse as per (20). **Supplementary Table S2** outlines the groups and challenges.

### *Shigella flexneri* challenge studies

*S. flexneri 2a* 2457T was streaked onto tryptic soy agar containing 0.025% Congo Red, incubated at 37°C overnight, and then subcultured into tryptic soy broth (TSB) for growth at 37°C until the absorbance at 600 nm (A_600_) reached 1.0. Bacteria were harvested by centrifugation, resuspended in PBS, and diluted to the desired concentration in a 30 µl volume for IN challenge. Mice were challenged with 1-10 x 10^6^ CFU per mouse of *S. flexneri* 2457T on Day 56 (four weeks after the final immunization). Mice were monitored twice a day for weight loss and health score for two weeks. Mice were euthanized when they lost more than 25% of their original weight for more than 72 h or their health score was considered poor and accompanied by a blood glucose level ≤100 mg/dL. All remaining mice were euthanized on Day 14 post-challenge.

### Antigen-specific IgG and IgA ELISAs

Fecal pellets and 100 μl blood obtained by the orbital sinus route were collected on Days 27, 41, and 55. Anti-IpaD and -IpaB IgG and IgA titers were determined as previously described with minor modifications (23). Briefly, microtiter plate wells were coated with 100 ng IpaB or IpaD in 100 µl PBS and incubated at 37°C for 3 h. Wells were blocked with 10% nonfat dry milk in PBS overnight. Sera were added to the wells in duplicates as the primary antibody for 2 h incubation at 37°C. After washing with PBS-0.05% Tween an HRP-secondary antibody (IgG(H+L), 1:1000; IgA, 1:500) was added and incubated for 1 h at 37°C. After an additional wash, OPD substrate (o-phenylenediamine dihydrochloride) was added and detected at 490nm by ELISA plate reader. Endpoint titers were determined by fitting antibody titrations to a five-parameter logistic model.

### Enumeration of IFN-γ or IL-17 secreting cells

Mouse necropsies in the IM studies were performed on Day 3 after challenge, with four mice from each group being sacrificed to collect, individually, the lung and spleen for immunology tests. Samples from mice in DBF with BECC438 alone *via* IN route study were collected on Day 14 for the mice that survived. Samples from mice in IN studies of BECC438 optimal formulations were collected on Day 52 from the pre-challenge vaccinated group. Mouse cells isolated from spleens and lungs were incubated for 24 h at 37°C in the presence of 5 µg/ml IpaB or IpaD in plates coated with antibodies against IFN-γ or IL-17 using a FluoroSpot assay as per the manufacturer’s specifications (Cellular Technology Limited). The cytokine-secreting cells were quantified using a CTL immunospot reader.

### Quantification of secreted cytokines after stimulation

Splenocytes and lung cells were incubated with 10 µg/ml IpaB, IpaD, or PBS for 48 h at 37°C. Supernatants were collected and analyzed with U-PLEX kits for cytokines: IFN-γ, IL-17A, IL-6, and TNF-α. Cytokine concentrations were determined using an MSD plate reader with associated analytical software (Meso Scale Discovery, Rockville, MD). While multiple cytokines were measured, the two that are focused on in this report are IL-17A and IFN-γ, as others did not show significant changes.

### Statistical analysis

Graphs were created using GraphPad Prism 9.0.1. Lung cytokine secretion was rescaled to the range between zero and one using min-max normalization [Y_normal_=(Y_origin_-Y_min_)/(Y_max_-Y_min_)]. Differences among unvaccinated (PBS) mice and antigen-vaccinated mice were analyzed using ANOVA. A *p*-value of less than 0.05 was considered significant for all comparisons. * *p*< 0.05; ** *p*<0.01; *** *p*< 0.001. For bacterial challenges, vaccinated groups were compared to PBS with Log-rank (Mantel-Cox) tests in GraphPad Prism.

## Results

### Intramuscular or intradermal immunization with DBF+BECC438 formulations does not protect mice against lethal *S. flexneri* challenge

We have previously demonstrated that IN delivery of DBF + dmLT protects against an otherwise lethal *S. flexneri* challenge (20). However, this formulation elicited poor protection when delivered IM. In this study, we wanted to determine whether bis-phosphorylated BECC438 could improve the protective efficacy of this formulation when delivered IM. BECC438 has been shown to be protective against *Yersinia pestis* and Influenza A when delivered IM with the appropriate antigen (26, 31). In our first experiment, mice were vaccinated IM with 0.1 to 40 μg DBF + 5 μg or 50 μg BECC438 (**Table 1**) or ID with 100, 250, or 500 ng DBF + 5 µg BECC438. As a positive control group, mice were vaccinated IN with 20 μg DBF + 2.5 μg dmLT. All the mice vaccinated IN with DBF + dmLT survived the otherwise lethal challenge, while all the mice vaccinated with PBS succumbed to the infection. Likewise, none of the groups vaccinated IM using BECC438 as the adjuvant demonstrated greater than 50% survival, with no significance detected between the groups (study of 5 μg BECC438: *p* > 0.066; study of 50 μg BECC438: *p* > 0.35). Similar results were found when the formulations with BECC438 were delivered ID, with each of the formulations providing <30% protection by this route (not shown).

**Table 1:** Vaccine efficacy in response to a DBF dose escalation with formulations containing either 5 or 50 µg BECC438.

DBF concentration	VE% of 5 µg BECC438	VE% of 50 µg BECC438
40 µg DBF	17	33
15 µg DBF	0	17
5 µg DBF	17	0
1.5 µg DBF	0	0
0.5 µg DBF	33	17
0.1 µg DBF	0	ND

Mice (n=6) were vaccinated IM with the indicated formulations. They were then challenged with 6 X 10^6^ CFU (in 30 µl) S. flexneri 2a 2457T. Vaccine Efficacy (VE) is shown where VE (%) = 1 – Attack Rate Vaccinated/Attack Rate Unvaccinated (PBS control) where all PBS vaccinated mice died. All mice vaccinated IN with 20 µg DBF + 2.5 µg dmLT survived and this was used as the positive control in this experiment.

To determine whether BECC438 admixed with DBF stimulated the T cell-related cytokine responses when administered *via* the IM route, the lungs from four of the surviving mice vaccinated with the DBF formulation were sampled early on Day 3 post-challenge (**Supplementary Figure S1**). It should be noted that by late afternoon of Day 3, all the remaining mice vaccinated with PBS had died. Secretion of IFN-γ and IL-17A from the harvested lung cells was then assessed following stimulation with IpaB and IpaD. In contrast to the prominent levels of IFN-γ and IL-17A secreted by lung cells from the DBF + dmLT IN group, the secretion of these cytokines was not observed for the lung cells from the mice vaccinated IM with DBF + BECC438 (**Supplementary Figure S1**). Thus, it appears that when delivered IM, BECC438 lacks the functional adjuvanticity needed to induce protective T cell immunity within the mouse lung against *S. flexneri* infection.

### IN immunization with BECC438 admixed with DBF induces only partial protection and elicits low levels of cytokines in splenocytes

To determine whether BECC438 could induce T cell responses *via* mucosal immunity, mice (n=10) were vaccinated IN with 20 µg DBF + 2.5 µg dmLT (positive vaccine control) or with 20 µg DBF + 5, 25, or 50 µg BECC438. All mice vaccinated with DBF formulations had similar levels of anti-IpaB or -IpaD IgG or IgA (**Supplementary Figure S2**). While 90% of mice vaccinated with the DBF + dmLT survived this challenge, the groups vaccinated with BECC438 had <40% survival (**Table 2**). It should be noted, however, that the surviving mice vaccinated with DBF + 25 or 50 µg BECC438 showed weight recovery trends similar to those of mice in the positive control group. This was not observed for the IM or ID administration groups (**Supplementary Figure S3**). Notably, the surviving mice in the IM vaccinated groups experienced delayed weight gain by at least 24 hours compared to the positive control groups. The weight recovery trends observed in the surviving mice vaccinated with DBF + 25 or 50 µg BECC438 indicated a positive outcome, suggesting that these vaccination regimens were effective in promoting recovery and restoring normal health parameters in the mice. To identify whether the improved weight recovery was from the adjuvanticity of BECC438 *via* the IN route and to investigate the BECC438 effects on cytokine levels, we assessed IL-17A, IFN-γ, IL-6, and TNF-α levels secreted upon stimulation of isolated lung cells and splenocytes from mice remaining on Day 14 post-challenge (**Supplementary Figure S4**). In this case, the lung cells did not provide a clear picture of what was occurring; therefore here, we report on the data obtained using the splenocytes. Furthermore, because all the mice vaccinated with PBS had died, we did not have a proper negative control to provide the usual statistical comparisons. Therefore, we compared the mice vaccinated with DBF + BECC438 with the positive control mice vaccinated with DBF + dmLT. After stimulation with IpaB or IpaD, high IL-17A and IFN-γ levels were detected in splenocyte supernatants from the mice vaccinated IN with dmLT as the adjuvant (**Supplementary Figure S4A**). When compared to these positive control mice, only the splenocytes from mice vaccinated with DBF + 25 µg or 50 µg BECC438 secreted similar IL-17A levels after stimulation with IpaB (*p* > 0.05); however, all three of the BECC438 groups secreted IFN-γ at levels similar to that of the positive control (*p* > 0.05). Conversely, when stimulated with IpaD, all BECC438 groups had statistically lower levels of IFN-γ and IL-17A than the positive control. Consistent with the IL-17A and IFN-γ levels, the levels of IL-6 and TNF-α secreted were similar to the positive control in all cases after stimulation with IpaB (**Supplementary Figure S4B**). When stimulating with IpaD, the 5 µg BECC438 group showed high levels of IL-6, which is consistent with the inability to regain weight at the rates seen for the other groups (**Supplementary Figure S4B**). Nevertheless, the protective efficacy of all the BECC438-treated groups was lower than for the DBF + dmLT vaccinated mice.

**Table 2:** Vaccine efficacy of 20 µg DBF with a BECC438 dose escalation administered by the IN route.

BECC438 concentration	VE%
50 µg BECC438	40
25 µg BECC438	30
5 µg BECC438	30

Mice (n=10) received IN vaccination using the specified formulations. Subsequently, they were exposed to a challenge of 1 X 10^7^ CFU of S. flexneri 2a 2457T, administered in 30 µl. Vaccine Efficacy (VE) is represented as a percentage, calculated using the formula VE (%) = 1 – (Attack Rate Vaccinated/Attack Rate Unvaccinated) where the control group receiving PBS did not survive. As a positive control, mice were IN vaccinated with 20 µg DBF + 2.5 µg dmLT, resulting in 90% survival in this specific challenge experiment.

### IN immunization with multimeric DBF + BECC438 formulated in an oil-in-water emulsion induces partial protection against *Shigella* infection

As mentioned above, subunit vaccines for use in humans are typically best presented using a multimeric antigen formulation. Therefore, because there were indications that DBF + BECC438 could elicit some level of protection relative to the negative control with cytokine responses that were on par in some cases with those seen for the positive control, we developed two oil-in-water formulations to boost the T cell responses to this antigen-adjuvant combination. To this end, squalene-based ME or NE was included as part of the IN formulations with 20 μg DBF and three distinct doses of BECC438 (**Table 3**). Regardless of the oil-in-water formulation used, the resulting anti-IpaB and anti-IpaD IgG and IgA titers were essentially equivalent (**Supplementary Figure S5**). All the mice vaccinated in the DBF + dmLT positive control survived the challenge, while all mice in the PBS vaccinated group succumbed to the challenge. The protective efficacies of mice vaccinated with DBF + 0.5 μg, 5 μg, and 50 μg BECC438 in the ME formulations were 60, 50, or 70%, respectively, with the DBF + 5 µg BECC438 in the ME group having somewhat slower weight recovery compared to other groups. In contrast, all the groups vaccinated with DBF + BECC438 formulated with NE had 40% or less survival (**Table 3**), suggesting that the aqueous phase of the oil-in-water emulsions can have an effect on formulation efficacy.

**Table 3:** Vaccine efficacy of 20 µg DBF with a BECC438 dose escalation in formulations containing ME or NE nanoemulsions after delivery *via* the IN route.

BECC438 concentration	VE%	
	ME	NE
50 µg BECC438	70	40
5 µg BECC438	50	30
0.5 µg BECC438	60	40

Mice (n=10) received IN vaccination using the specified formulations. Following vaccinations, they were challenged with 1 X 10^7^ CFU of S. flexneri 2a 2457T (in 30 µl). The Vaccine Efficacy (VE) is calculated as VE (%) = 1 – (Attack Rate Vaccinated/Attack Rate Unvaccinated), with the unvaccinated group receiving PBS serving as the control. All the mice in the PBS group succumbed to the challenge. In this experiment, the positive control consisted of IN vaccination with 20 µg DBF + 2.5 µg dmLT, resulting in a remarkable 100% survival rate.

Because some of the mice did not tolerate DBF + 50 µg BECC438 ME nanoemulsion formulation particularly well (30% of mice suffered from illness during these vaccinations), we reduced the BECC438 to 10 µg to better control the overall immunostimulatory potential. At the same time, other ongoing studies within our laboratory had shown potential protective efficacy with the use of chitosan prepared with the mast cell-activating adjuvant C48/80 (manuscript in preparation and (32)), which we call Chi-C48/80. This led us to consider this as a nanoparticle formulation alongside the ME nanoemulsion. Furthermore, prior to the completion of these studies, we developed a self-adjuvanting form of DBF called L-DBF, which is a genetic fusion of LTA1 and DBF that protects mice as well as admixed DBF + dmLT (20). We, therefore, used L-DBF as the positive control for these studies moving forward. When we vaccinated mice with L-DBF or 20 µg DBF + 10 µg BECC438 admixed with either ME or with Chi-C48/80, we found that the resulting anti-IpaB and anti-IpaD IgG and IgA titers were essentially equivalent (**Supplementary Figure S6**). In this experiment, the mice vaccinated with L-DBF had 80% survival following a high dose challenge, while none of the mice vaccinated with PBS survived. Unfortunately, the reduction of BECC438 to 10 µg lowered the protective efficacy to 30%, and none of the mice from the Chi-C48/80 group survived the challenge.

### Optimized BECC438 formulations can elicit IL-17 and IFN-γ secretion in lung cells when administered IN

**Figure 1:**
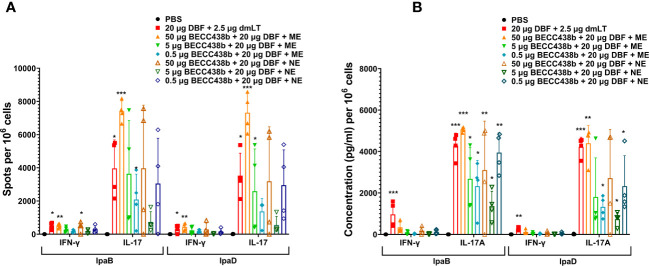
IL-17A and IFN-γ secretion from isolated lung cells from mice vaccinated with DBF + BECC438 with ME or NE. All samples were collected from mice 3 days before a parallel group was challenged. **(A)** Single lung cell suspensions were prepared and used to assess antigen-specific IL-17 and IFN-γ secreting cells after antigen stimulation. Cells were stimulated with 10 µg IpaB (left) or IpaD (right). IL-17 and IFN-γ secreting cells were enumerated by ELISpot and are presented here as spot-forming cells/10^6^ total cells. **(B)** Single lung cell suspensions were stimulated with 10 µg IpaB (left) or IpaD (right). Cytokine levels in the cell supernatants were then determined by Meso Scale Discovery analysis as per the manufacturer’s specifications and are presented here as pg/ml/10^6^ cells. Data were plotted as actual values from individuals ± SD (n = 4) in each group. Significance was calculated by comparing groups that were unvaccinated (PBS) and mice vaccinated with antigens using the Welch t-test. *p<0.05; **p<0.01; ***p< 0.001.

**Figure 2:**
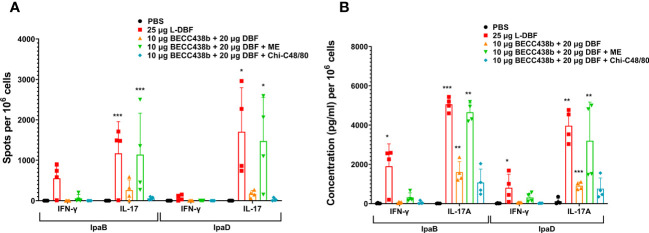
IL-17 and IFN-γ secretion from isolated lung cells from mice vaccinated with DBF + BECC438 alone, with ME, or with Chi-C48/80. All samples were collected three days prior to a parallel group being challenged. **(A)** Lung cell suspensions were prepared, and the antigen-specific IL-17 and IFN-γ secreting cells were assessed. Cells were stimulated with 10 µg IpaB (left) or IpaD (right). The quantification of IL-17 and IFN-γ secreting cells was determined using ELISpot, and the results are presented as spot-forming cells/10^6^ total cells. **(B)** Following the preparation of single cell suspensions, cells were stimulated with 10 µg IpaB (left) or IpaD (right). The levels of cytokines in the cell supernatants were measured using Meso Scale Discovery analysis, according to the manufacturer’s specifications. The results are presented as pg/ml/10^6^ cells. Data were plotted as actual values from individuals ± SD (n = 4) in each group. Statistical significance was determined by performing the Welch t-test, comparing the unvaccinated group (PBS) with the mice vaccinated with antigens. *p<0.05; **p<0.01; ***p<0.001 indicate the level of significance.

To understand the potential reasons for the low efficacy of these BECC438-containing formulations relative to DBF + dmLT or L-DBF positive controls, we assessed the T cell-related cytokines elicited by the ME, NE, and Chi-C48/80 formulations. In the first trial, the frequency of IFN-γ and IL17-secreting cells were enumerated from mice vaccinated with either the ME or NE formulations (**Figure 1A**). The frequencies of the IFN-γ- and IL-17- secreting T cells from the lungs of mice vaccinated with DBF + dmLT and the 20 μg DBF + 50 μg BECC438 with ME groups were significantly increased following stimulation with IpaB and IpaD. When the levels of secreted cytokines after stimulation were assessed, high levels of IL-17A were secreted from lung cells from most of the groups (**Figure 1B**), but only the DBF + dmLT group showed significant IFN-γ secretion. In the second trial, 25 µg L-DBF was used as the positive control for comparison with 20 μg DBF + 10 μg BECC438 alone, with ME or with Chi-C48/80, and the frequency of lung IFN-γ- and IL-17-secreting cells was quantified after stimulation with IpaB and IpaD (**Figure 2A**). The frequency of IL-17 secreting cells from the L-DBF group and the 20 μg DBF + 10 μg BECC438 with ME were significantly higher compared to the PBS group after stimulation by IpaB and IpaD. In contrast, none of the groups had a higher frequency of IFN-γ-secreting cells compared to the PBS group (25 µg L-DBF vs. PBS: IpaB IFN-γ *p*=0.63). In contrast, when the levels of cytokines secreted after stimulation of the lung cells with IpaB or IpaD were assessed, only the Chi-C48/80 failed to show a significantly higher level of IL-17A secretion (**Figure 2B**), and only the L-DBF group showed significantly greater IFN-γ secretion compared to PBS group. Thus, as we have previously described (20), the only groups within these two sets of experiments that contained the LTA1, either in the form of dmLT or as L-DBF, showed significant levels of secreted IL-17A and IFN-γ upon stimulation with both IpaB and IpaD. On the other hand, BECC438 + DBF in the context of the ME nanoemulsion demonstrated strong IL-17 responses after stimulation with IpaB and IpaD. Meanwhile, the Chi-48/80 group failed to show elevated IL-17 and IFN-γ in any of the cases, suggesting that BECC438 + DBF is best presented as part of an oil-in-water nanoemulsion.

### The presence of LTA1 fused with DBF enhances the protection seen for the optimized BECC438 formulations by eliciting greater cytokine responses

To increase the protective efficacy against the intracellular pathogen *Shigella*, L-DBF (instead of DBF) was used as a component of the BECC438 with ME formulation. Because of the high efficacy of 25 µg L-DBF alone, we believed that BECC438 might allow for it to be used in dose- and antigen-sparing amounts or with fewer immunizations required. Two separate experiments were performed here, with one using 1, 10, or 15 μg L-DBF + 10 μg BECC438 with ME in a prime-boost-boost regimen and another using 0.5 μg L-DBF + 1 μg BECC438 with ME in prime only, prime-boost, or prime-boost-boost regimens. In the first L-DBF dose escalation study, >90% of mice vaccinated with any of the L-DBF doses survived (**Table 4**) with similar IgG and IgA levels (**Supplementary Figure S7**). However, we have observed that the administration of 10 μg of BECC in combination with either 15 μg or 10 μg of L-DBF in ME induced certain side effects, which indicated that 1 μg L-DBF + 10 μg BECC438 with ME would be a considerable formulation. The second study used a lower BECC438 and L-DBF dose that could still afford protection to assess the number of boosts required for protection. The group given two boosters demonstrated 60% protection, while the single boost group was afforded 50% protection. The anti-IpaB IgG levels were similar for the one and two boost groups, while the anti-IpaB IgA for both were either below or just above (at the second boost) baseline (**Supplementary Figure S8**). In contrast, the anti-IpaD IgG levels of the single boost group were much lower than the group with two boosts with the anti-IpaD IgA being very low, as was seen with the anti-IpaB IgA. Unfortunately, the prime administration alone did not induce an immune response sufficient to protect against a subsequent challenge (**Table 5**). For this group, IgA was not above the baseline level and the IgG levels were much lower than the prime-boost-boost group. Only the anti-IpaB IgG levels approached that of the groups with boosters. Ultimately, it was found that BECC438 with ME allowed for the use of lower doses of L-DBF to provide protection (down to 1 µg), but that optimal protection still required a prime-boost-boost immunization schedule.

**Table 4:** Vaccine efficacy for an L-DBF dose escalation formulated with BECC438 and ME after IN administration.

Formulation	VE%
10 µg BECC438 + 15 ug L-DBF +ME	90
10 µg BECC438 + 10 ug L-DBF +ME	100
10 µg BECC438 + 1 ug L-DBF +ME	90

Mice (n=10) were vaccinated IN with the indicated formulations. They were then subjected to 1 X 10^6^ CFU (in 30 µl) S. flexneri 2a 2457T. Vaccine Efficacy (VE) is shown where VE = 1 – Attack Rate Vaccinated/Attack Rate Unvaccinated (PBS control) where the control group receiving PBS vaccination witnessed mortality in all mice. Positive control mice vaccinated IN with 25 µg L-DBF where all the mice survived.

**Table 5:** Vaccine efficacy of L-DBF formulated with BECC438 and ME delivered *via* IN route using different booster regimens.

Formulation	Booster	VE%
1 µg BECC438 + 0.5 ug L-DBF +ME	2	60
1 µg BECC438 + 0.5 ug L-DBF +ME	1	50
1 µg BECC438 + 0.5 ug L-DBF +ME	0	30

A total of 10 mice in each group were intranasally (IN) vaccinated using the specified formulations. Subsequently, they were exposed to a challenge of 1 X 10^6^ CFU (in 30 µl) of S. flexneri 2a 2457T. The Vaccine Efficacy (VE) is depicted as VE (%) = 1 – (Attack Rate Vaccinated/Attack Rate Unvaccinated), with the control group receiving PBS experiencing mortality among all mice. Notably, the positive control mice were IN vaccinated with 25 µg L-DBF, resulting in the survival of all mice following the challenge.

**Figure 3:**
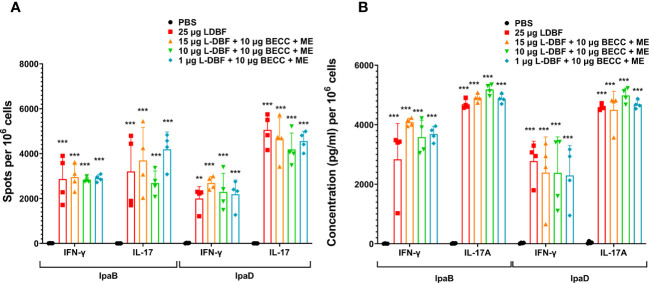
IL-17 and IFN-γ secretion from isolated lung cells from mice vaccinated with a dose escalation of L-DBF in a formulation with BECC438 + ME. All samples were collected three days before a parallel group was challenged. **(A)** To assess antigen-specific IL-17 and IFN-γ secreting cells, single lung cell suspensions were prepared. Cells were stimulated with 10 µg IpaB (left) or IpaD (right). IL-17 and IFN-γ secreting cells were performed by ELISpot and are presented here as spot-forming cells/10^6^ cells. **(B)** Single cell suspensions were then stimulated with 10 µg IpaB (left) or IpaD (right) and the levels of cytokines in the cell supernatants were determined by Meso Scale Discovery analysis following the manufacturer’s specifications and are presented here as pg/ml/10^6^ cells. The data are displayed as the actual values obtained from individual samples, accompanied by the mean ± SD (n = 4) for each group. Significance was calculated by comparing groups that were unvaccinated (PBS) and mice vaccinated with antigens using the Welch t-test. **p<0.01; ***p< 0.001.

**Figure 4:**
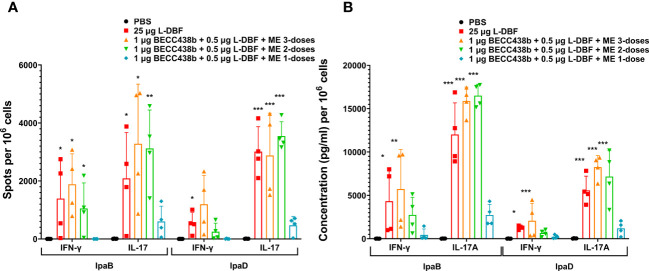
IL-17 and IFN-γ secretion from isolated lung cells from mice vaccinated with BECC438 + L-DBF + ME using distinct booster regimens. All samples were collected 3 days before a parallel group of mice were challenged. **(A)** The enumeration of IL-17 and IFN-γ secreting cells specific to the antigen was conducted using ELISpot on single lung cell suspensions. Stimulation with 10 µg of IpaB (left) or IpaD (right) was employed for this purpose. The resulting spot-forming cells/10^6^ total cells were recorded and analyzed. **(B)** The measurement of cytokine levels in the cell supernatants was performed using Meso Scale Discovery analysis, following the manufacturer’s specifications. Single cell suspensions were stimulated with 10 µg of IpaB (left) or IpaD (right), and the cytokine levels were quantified as pg/ml/10^6^ cells. Data were plotted as actual values from individuals ± SD (n = 4) in each group. The significance of the results was assessed using the Welch t-test, comparing the unvaccinated group (PBS) with the group of mice vaccinated with antigens. Significance levels are denoted as *p<0.05, **p<0.01, and ***p<0.001. .

To understand the effects of these modified formulations on the cellular immune response, we assessed their effect on the IFN-γ and IL17 cytokine responses of isolated lung cells. In prior work, we found that 10 μg L-DBF or 1 μg L-DBF alone does not protect mice against *Shigella* infection and elicits lower IFN-γ and IL-17 responses when compared to 25 μg L-DBF (20). This lack of protection contrasts with what is seen when these doses of L-DBF are formulated with BECC438 and ME (**Table 4**). We, therefore, chose to assess the frequency of IFN-γ- and IL17-secreting cells from the lung upon antigen stimulation following vaccination using the dose escalation and the booster regimens described above (**Figures 3A**, **4A**). After stimulating with IpaB and IpaD, all L-DBF + BECC438 with ME vaccinated groups from the dose escalation study groups, regardless of concentration, induced significantly higher frequencies of IFN-γ or IL17 secreting cells compared to those from the PBS group (**Figure 3A**). In contrast, in the prime-boost regimen study, only the positive control and prime-boost-boost groups had significantly higher frequencies of IFN-γ and IL17 secreting cells after stimulation than the PBS control, though the prime-boost regimen did give rise to an elevated frequency of IL17 secreting cells after stimulation with either IpaB or IpaD (**Figure 4**). Interestingly, while there was an increase in the frequency of IFN-γ secreting cells from the prime-boost lung cells when they were stimulated with IpaB, no significance was seen in the frequency of IFN-γ cells after stimulation with IpaD (**Figure 4A**). When the levels of secreted IFN-γ and IL-17A were quantified for the lung cells after stimulation, all groups vaccinated with L-DBF, regardless of concentration, had significantly higher levels of both IFN-γ and IL-17A than the lung cells from the PBS group (**Figure 3B**). In contrast, only the positive control and prime-boost-boost groups had significantly higher levels of both cytokines. The results suggest that even low levels of LTA1 on L-DBF can enhance the stimulation of Th1 and Th17 cytokines in the presence of BECC438. This is especially true for the increased secretion of IFN-γ, which is necessary for a protective response against *Shigella* spp (20, 33).

### Correlation of protection with the secretion of IFN-γ and IL-17 after stimulating with IpaB or IpaD

**Figure 5:**
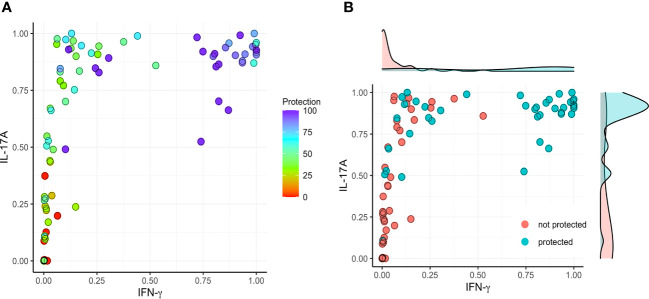
Correlation between protection and the secretion of IFN-γ and IL-17 after stimulating with IpaB. The original data (cytokine levels from **Figures 1**–**4**) were rescaled (normalized) into the [0, 1] range using Y_normal_=(Y_origin_-Y_min_)/(Y_max_-Y_min_). **(A)** The normalized data were then used to establish a model comparing IFN-γ (X) and IL-17A (Y) with protection (left; color map). **(B)** A >60% vaccine efficacy was used as a targeted cut-off value (right).

**Figure 6:**
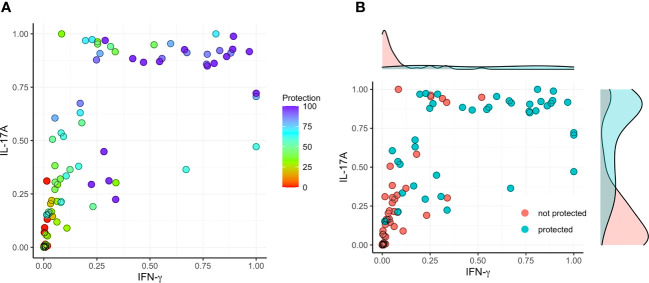
Correlation between protection and the secretion of IFN-γ and IL-17 after stimulating with IpaD. The initial cytokine data (from **Figures 1**–**4**) underwent rescaling (normalization) to fit within the [0, 1] range using the formula Y_normal_=(Y_origin_-Y_min_)/(Y_max_-Y_min_). **(A)** The normalized data was subsequently utilized to construct a model, correlating IFN-γ (X) and IL-17A (Y) with the degree of protection (left; color map). **(B)** A threshold of >60% vaccine efficacy was employed as the designated cut-off value (right).

**Figure 7:**
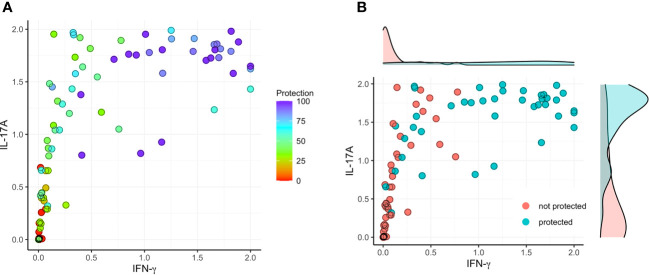
Correlation between protection and the sum of secreted IFN-γ or IL-17 after stimulation with IpaB or IpaD. The raw cytokine data (as depicted in **Figures 1**–**4**) underwent a transformation process to fit within the normalized range of [0, 1]. **(A)** The normalized data were used to establish a model that involved the sum (ranging from 0 to 2) of IFN-γ stimulating with IpaB or IpaD (X; IFN-γ sum = IFN-γ of IpaB + IFN-γ of IpaD) and the sum (ranging from 0 to 2) of IL-17A stimulating with IpaB or IpaD (Y; IL-17A sum = IL-17A of IpaB + IL-17A of IpaD) with protection (left; color map). **(B)** To determine a threshold for significant vaccine efficacy, a cut-off value of >60% was selected (right).

Based on the findings presented here, we evaluated the correlation between vaccine-mediated protection with the secretion of IFN-γ and IL-17A by lung cells following stimulation with IpaB or IpaD. We first normalized the data into a 0 to 1 scale and established a model involving IFN-γ (X) and IL-17A (Y) versus protection (**Figures 5**–**7**). IL-17 stimulated by IpaB was a strong predictor that mice would survive an otherwise lethal challenge with *Shigella* (**Figure 5**); however, IpaB-induced IL-17 secretion alone did not appear to predict the highest level of survival in the challenge. In general, IpaB induction of IFN-γ secretion by lung cells (>0.5) appeared to be required to reach >60% protection. IpaD induction of IL-17 and IFN-γ secretion also showed a significant positive correlation with protection, albeit this correlation was lower than for IpaB, and both cytokines were required to provide a successful immunization (**Figure 6**). We also added the normalized outcomes seen for IpaB and IpaD together and established a correlation curve between these cytokines and the vaccine-mediated protection (**Figure 7**). In general, the cut-off point for the cytokine level to predict a protective outcome was ~1.0, which suggests that a successful vaccine candidate should be able to induce both IL-17 and IFN-γ and that both IpaB and IpaD contribute to this outcome.

## Discussion

Shigellosis can be fatal for infants and children under five years of age, especially for those living in low-income countries where it is endemic and there is limited access to basic life-saving treatments and sanitation infrastructure (34). Existing and emerging antibiotic resistance among the shigellae calls for an effective vaccine against shigellosis, but none have been licensed to date. A broadly protective vaccine as part of an optimized formulation tailored for use in humans is urgently needed. We previously tested subunit vaccine candidates in which IpaD and IpaB of the *Shigella* T3SS were genetically fused (DBF) using the mouse lethal pulmonary model and found that, when administered IN with dmLT or as a fusion that contains LTA1 moiety of dmLT (L-DBF), they have a high protective efficacy (15, 20). Additionally, due to the conservation of IpaD and IpaB across different *Shigella* species, our vaccine candidates could also induce a cross-protective immune response against multiple *Shigella* serotypes. Unfortunately, monomeric antigens tend to be poor at inducing strong protective responses in humans and a multimeric presentation is often required. We, therefore, chose to explore the use of different formulations (ME, NE, and Chi-C48/80) with our DBF and L-DBF vaccine candidates to identify potential presentation and adjuvant combinations that could be of use in developing a human vaccine against *Shigella*. ME is an emulsion-based system with small droplet sizes (≈ 140 nm), which improves antigen stability, uptake, and immunogenicity, while Chi-C48/80 is a chitosan derivative that acts as an adjuvant that enhances immune responses through its interactions with immune cells. The new squalene-based emulsion NE had been shown to have a smaller average size (≈ 65 nm) compared to ME. In previous studies, we have investigated the adjuvant effects and physical properties of ME and Chi-C48/80 in the context of enhancing the immune response against *Salmonella* and *Pseudomonas* infections (32, 35). In this study, ME proved to be a more effective adjuvant for enhancing the immune response against *Shigella* infection. We found NE and Chi-C48/80 did not demonstrate the same level of adjuvant activity as ME.

In previous work, we tested multiple delivery routes and found that IN administration best induced a mucosal immune response that protected mice from lethal challenge (14, 15), though a certain degree of protection could be elicited by IM and ID administration (36, 37). Here, we tested the IM, ID, and IN routes by formulating DBF with BECC438, a novel bisphosphorylated lipid A adjuvant that is a TLR4 agonist and a biosimilar of MPLA (26, 30), which is approved for use in some human vaccines. When DBF at different concentrations was administered IM and ID with BECC438, little protection was seen except for with IM at the highest DBF concentrations (**Table 1**). In contrast, DBF with BECC438 delivered IN provided some protection, though not as much as DBF with dmLT. In this case, however, IN administration elicited IL-17 and IFN- γ responses, suggesting that it did promote responses deemed necessary for protection against *Shigella* (**Table 2**, **Supplementary Figure S1**). We, thus, chose to reformulate the DBF with BECC438 to present the antigen in a polymeric form by including candidate oil-in-water nanoemulsions and nanoparticles (chitosan). While the chitosan formulation failed to provide protection, the nanoemulsions greatly increased the protection of mice against otherwise lethal *Shigella* challenge (**Table 3**). This was especially true for the ME nanoemulsion, which is similar to the human-approved AS03 adjuvant formation with BECC438 replacing the α-tocopherol adjuvant component of the formulation. While we could have included Alhydrogel (an aluminum salt) as an adjuvant/carrier, we felt that the nanoemulsions with their small size (100 to 200 nm) were a better fit for IN administration, though there are not yet any such formulations for administration by this route.

The highest dose of BECC438 enhanced the adjuvanticity of the ME oil-in-water emulsion the best; however, we were somewhat concerned that too much adjuvant could overstimulate the host’s innate immune system and potentially lead to inflammation. To reduce the total amount of adjuvant present, we combined low doses of the self-adjuvanting L-DBF with the BECC/ME formulation instead of DBF so that we could reduce the amount of BECC present. Our previous work has demonstrated that L-DBF alone provides cross-protection against lethal challenge by different *Shigella* serotypes; however, this required a dose of at least 25 µg (15, 20). When BECC438 was used together with the LTA1 moiety of L-DBF, it was found to provide protection at a much lower dose (**Table 4**); however, it was also found that the L-DBF needed for protection could be reduced to 1 µg. Neither the 10 µg or 1 µg L-DBF doses could elicit protection on their own (20). The inclusion of BECC438 with ME in these L-DBF doses caused the Vaccine Efficacy (VE) to increase significantly from 10% to 90%-100%. Interestingly, the BECC438 and L-DBF formulated with ME could even be reduced to 1 µg and 0.5 µg, respectively, and still provide a VE of 60% with a prime-boost-boost regimen and 50% VE with a prime-boost regimen (**Table 5**); however, protection dropped off sharply for a prime vaccination without any booster vaccinations.

Early work on *Shigella* vaccine development mainly focused on antibodies, particularly mucosal neutralizing IgA, which directly prevents the bacteria from invading mucosal epithelial cells. It has been proposed that mucosal IgA levels correlate with the vaccine effects. Although antibodies are important for pathogen clearance, the data presented here indicate that T cell responses raised during vaccination are also important and it has been reported that induction of IL-17 and IFN-γ responses in response to *Shigella* infection also have a role in pathogen clearance and protection of the host (16). Our earlier studies suggested that host protection against a lethal *S. flexneri* challenge is dependent upon strong IFN-γ and IL-17 responses in a mouse model. IFN-γ is an important cytokine related to the Th1 response, while IL-17 is a major cytokine that is part of the Th17 response. Therefore, in this study, we analyzed the correlation between Th1/Th17-related cytokines with the vaccine efficacy. We found that vaccine-induced IL-17 secretion was an important component of protective immunity against *Shigella* infection, but IFN-γ was also found to be important. Such a correlation was not found for other cytokines, e.g., TNF-α (not shown).

In conclusion, our findings suggest that a Th1/Th17 response induced by vaccines using BECC438 as an adjuvant could contribute to protection from *Shigella* challenge and this effect could be seen at low BECC438 and antigen doses when LTA1 was also present (as a component of L-DBF). The enhanced BECC438 and L-DBF, as part of a polymeric presentation with the ME nanoemulsion, also suggests that such a formulation could be suitable for use in humans. Some reactivity was observed when BECC438 and LTA1 (as part of L-DBF) were both used at high doses, most likely due to there being too much adjuvant power acting upon innate immune responses. However, the presence of both adjuvants (BECC438 and LTA1) at low doses did not induce any ill effects in the mice and the ability to introduce this formulation at a mucosal site (intranasally) ensured that a proper mucosal immune response was induced that was safe and protective against lethal *Shigella* challenge.

## Data availability statement

The original contributions presented in the study are included in the article/**Supplementary Material**. Further inquiries can be directed to the corresponding author.

## Ethics statement

The mouse animal protocols were reviewed and approved by the University of Kansas Institutional Animal Care and Use Committee Practices (protocol AUS 222-01). The study was conducted in accordance with the local legislation and institutional requirements.

## Author contributions

WLP was responsible for conceptualization, acquiring financial support, project administration, editing of the original manuscript draft and overall supervision; TL was responsible for data curation, initial data analysis, data acquisition, method development and writing of the initial manuscript draft; SD was responsible for assisting with data acquisition, and immunological method development; DH was responsible for assisting with data acquisition, data analysis, immunological method development and reviewing of the manuscript; AJ was responsible for the purification of protein antigens, protein analysis, formulation and methods development related to protein purification; GH prepared the formulations and biophysical characterization (quality control) of the BECC438 and ME; ZD was responsible for the collection of specimens, protein purification, and animal method development; QZ was responsible for collection of specimens and animal method development; SW was responsible for in-depth statistical analyses and experimental design; DV prepared BECC438 and contributed to its biophysical analysis; RE critically read the paper, provided the BECC438 and help to edit final versions of the manuscript; WDP was responsible for conceptualization and final manuscript editing and writing. All authors contributed to the article and approved the submitted version.
