# Supplementary material for: Impact of the TLR4 agonist BECC438 on a novel vaccine formulation against Shigella spp
Source: Front Immunol. 2023 Sep 6;14:1194912. doi: 10.3389/fimmu.2023.1194912 (PMC10512073; doi:10.3389/fimmu.2023.1194912)
Supplement: Supplementary file 1 [file DataSheet_1.pdf]

Supplementary Table S1. Acronyms used in this paper.

T3SS	Type III secretion system
DBF	IpaD-IpaB fusion protein
dmLT	Double-mutant heat-labile enterotoxin
LTA1	A1 moiety of the active subunit of dmLT
L-DBF	LTA1 fusion with DBF
BECC	Bacterial Enzymatic Combinatorial Chemistry
ETEC	Enterotoxigenic *Escherichia coli*
IN	Intranasal
IM	Intramuscular
ID	Intradermal
TLR-4	Toll-like receptor 4
LDAO	Lauryl-dimethylamine oxide
ME	MedImmune emulsion
NE	Newly developed Squalene-based emulsion
MOPS	3-(N-morpholino)propanesulfonic acid
Chi-C48/80	Adjuvant compound 48/80 loaded chitosan
HRP	Horseradish peroxidase
OPD	O-phenylenediamine dihydrochloride
MPLA	Monophosphoryl lipid A.

Supplementary Table S2. Outline of experimental groups.

Title of Results section	Groups[1]	Route	Challenge[2] (*S. flexneri 2457t)*
Intramuscular or intradermal immunization with DBF+BECC438 formulations does not protect mice against lethal *S. flexneri* challenge.	20 µg DBF + 2.5 µg dmLT	IN	6×10^6 CFU
	5 µg BECC438 + 100 ng DBF	ID	
	5 µg BECC438 + 250 ng DBF	ID	
	5 µg BECC438 + 500 ng DBF	ID	
	20 µg DBF + 2.5 µg dmLT	IN	6×10^6 CFU
	5 µg BECC438 + 40 µg DBF	IM	
	5 µg BECC438 + 15 µg DBF	IM	
	5 µg BECC438 + 5 µg DBF	IM	
	5 µg BECC438 + 1.5 µg DBF	IM	
	5 µg BECC438 + 0.5 µg DBF	IM	
	5 µg BECC438 + 0.1 µg DBF	IM	
	20 µg DBF + 2.5 µg dmLT	IN	6×10^6 CFU
	50 µg BECC438 + 40 µg DBF	IM	
	50 µg BECC438 + 15 µg DBF	IM	
	50 µg BECC438 + 5 µg DBF	IM	
	50 µg BECC438 + 1.5 µg DBF	IM	
	50 µg BECC438 + 0.5 µg DBF	IM	
	50 µg BECC438 + 0.1 µg DBF	IM	
IN immunization with BECC438 admixed with DBF induces only partial protection and elicits low levels of cytokines in splenocytes.	20 µg DBF + 2.5 µg dmLT	IN	1×10^7 CFU
	50 µg BECC438 + 20 µg DBF	IN	
	25 µg BECC438 + 20 µg DBF	IN	
	5 µg BECC438 + 20 µg DBF	IN	
IN immunization with multimeric DBF + BECC438 formulated in an oil-in-water emulsion induces partial protection against *Shigella* infection.	20 µg DBF + 2.5 µg dmLT	IN	1×10^7 CFU
	50 µg BECC438 + 20 µg DBF + ME	IN	
	5 µg BECC438 + 20 µg DBF + ME	IN	
	0.5 µg BECC438 + 20 µg DBF + ME	IN	
	50 µg BECC438 + 20 µg DBF + NE	IN	
	5 µg BECC438 + 20 µg DBF + NE	IN	
	0.5 µg BECC438 + 20 µg DBF + NE	IN	
Optimized BECC438 formulations can elicit IL-17 and IFN-γ secretion in lung cells when administered IN.	25 µg L-DBF	IN	1×10^6 CFU
	10 µg BECC438b + 20 µg DBF	IN	
	10 µg BECC438b + 20 µg DBF + ME	IN	
	10 µg BECC438b + 20 µg DBF + Chi-C48/80	IN	
The presence of LTA1 fused with DBF enhances the protection seen for the optimized BECC438 formulations by eliciting greater cytokine responses.	25 µg L-DBF	IN	1×10^6 CFU
	10 µg BECC438 + 15 ug L-DBF +ME	IN	
	10 µg BECC438 + 10 ug L-DBF +ME	IN	
	10 µg BECC438 + 1 ug L-DBF +ME	IN	
	25 µg L-DBF	IN	1×10^6 CFU
	1 µg BECC438 + 0.5 ug L-DBF +ME	IN	
	1 µg BECC438 + 0.5 ug L-DBF +ME* (1 boosters)	IN	
	1 µg BECC438 + 0.5 ug L-DBF +ME* (0 boosters)	IN	

[1] All studies followed the immunization procedure of one primer + two boosters except the groups labeled with *.

[2] in 30 µl per mouse.

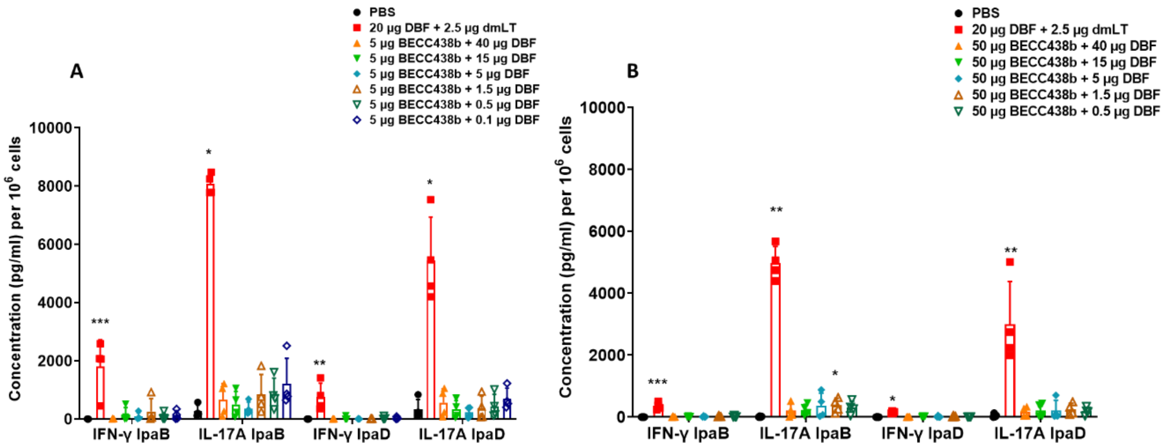

Supplementary Figure S1. IL-17A and IFN-γ secretion from lung cells prepared from mice vaccinated intramuscularly (IM) with 5 µg (A) or 50 µg (B) BECC438b + DBF on Day 3 after challenge. All samples were collected on day 3 after the challenge. Single-cell lung suspensions were incubated with 10 µg IpaB and IpaD. Cytokine levels were determined by Meso Scale Discovery analysis as per the manufacturer's specifications and are presented here as pg/ml/10^6 cells. Secretion of IFN-ɣ and IL-17A was noted as a response of either IpaB or IpaD stimulation. Data were plotted as actual values from individuals ± SD (n = 4) in each group. Significance was calculated by comparing groups that were unvaccinated (PBS) and mice vaccinated with antigens using a Welch t-test. *p<0.05; **p<0.01; ***p< 0.001.

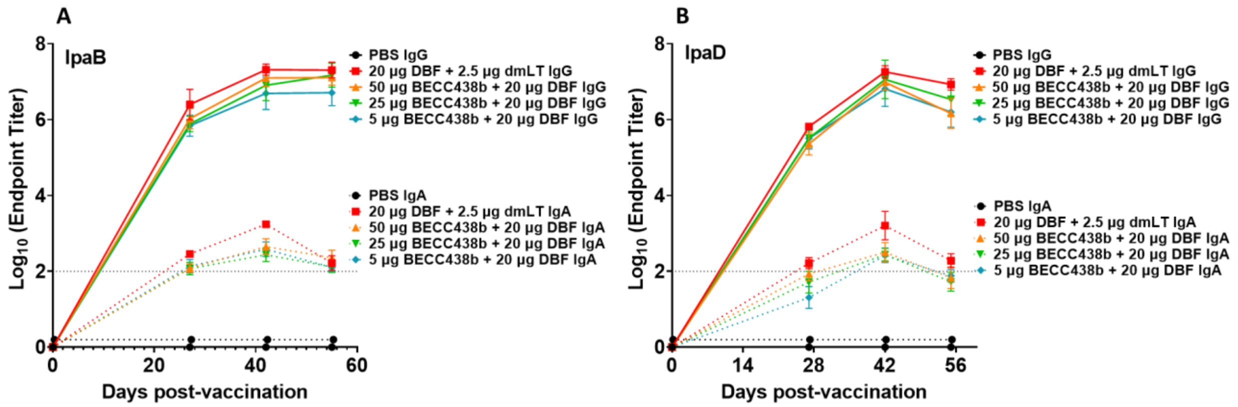

Supplementary Figure S2. Antigen-specific IgG and IgA responses. Mice were vaccinated intranasally (IN) with PBS, 20 µg DBF+2.5 µg dmLT, 50 µg, 25 µg or 5 µg BECC438b + 20 µg DBF three times (Day 0, 14 and 28). Blood and fecal samples were collected and serum titers for serum IgG (filled symbols with solid lines) and fecal IgA (open symbols with dashed lines) specific for IpaB (A) or IpaD (B) were measured by ELISA. The individual titers are represented as EU ml^{-1}. Each point represents the mean and error bars represent SD of each group (n=10/group).

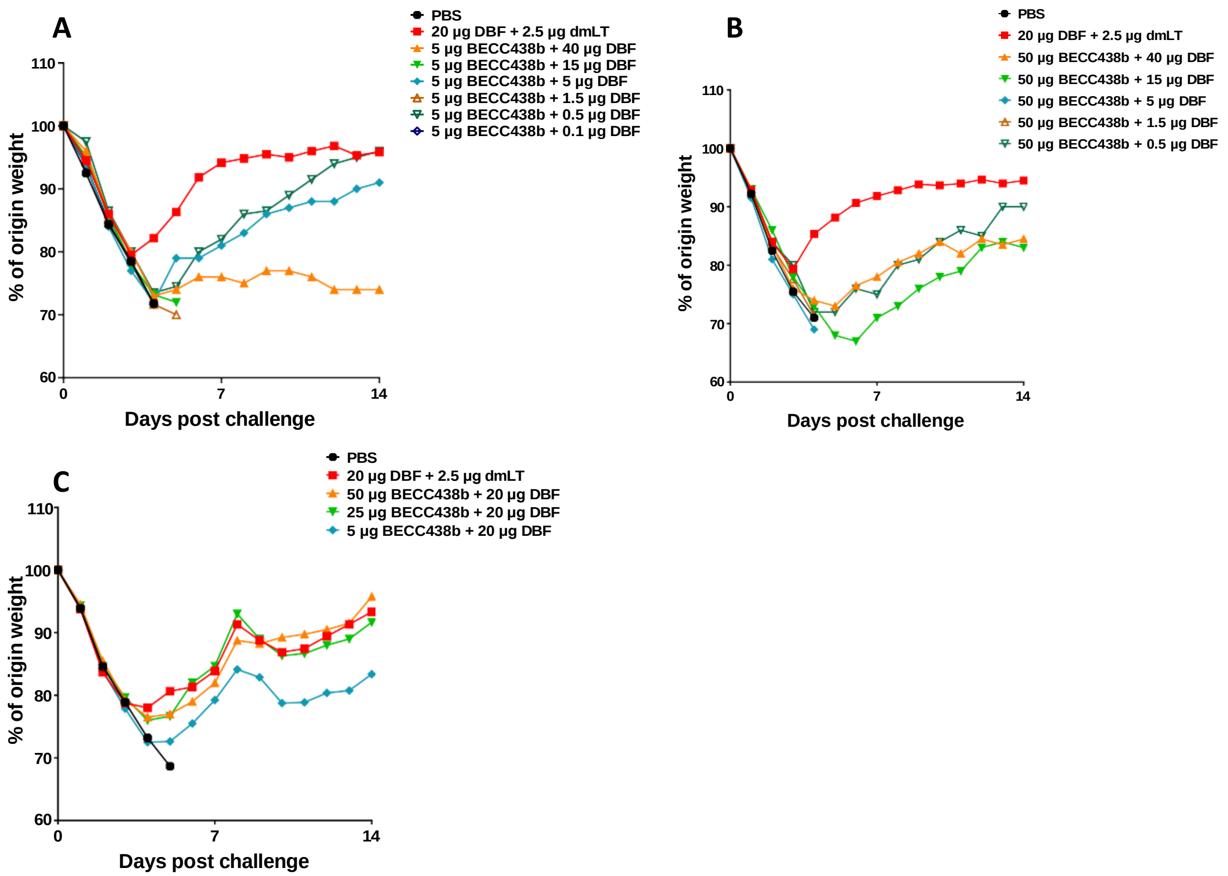

Supplementary Figure S3. The weight loss of mice following *Shigella* challenge. Mice vaccinated with different formulations via IM (Panel A & B) or IN (Panel C) routes were then challenged IN with 6 x 10^6 CFU per mouse of *S. flexneri* 2a and their weights monitored every 24 h for 14 days.

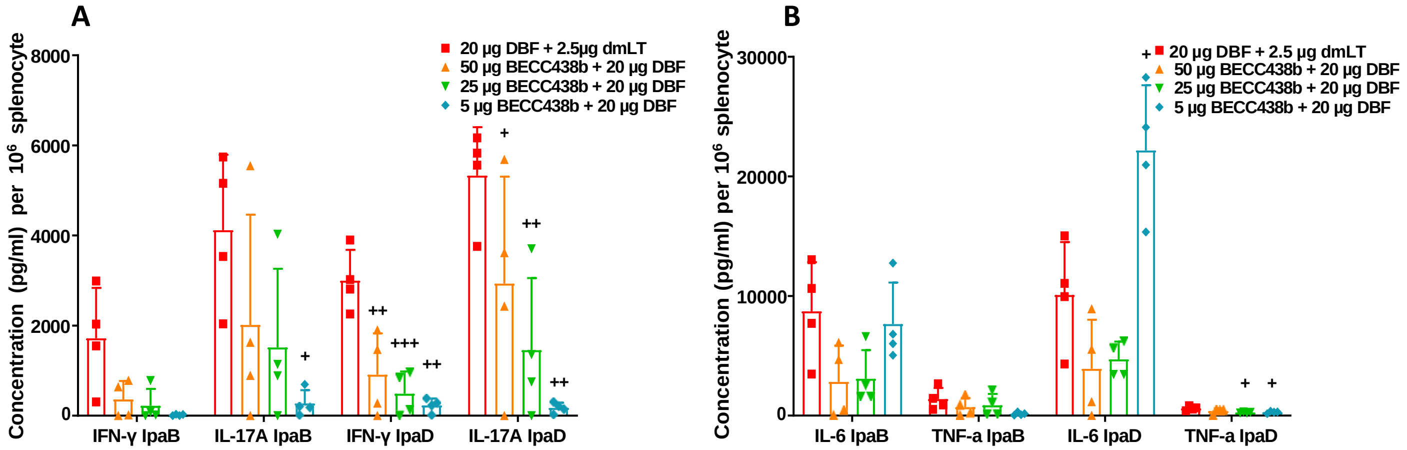

Supplementary Figure S4. All samples were collected on day 14 after the challenge. The single- cell splenocyte suspensions were used to assess antigen-specific IFN-ɣ and IL-17A (A), IL-6 and TNF-α (B) secretion from spleen cell suspensions. Cells were incubated with 10 µg IpaB or IpaD. Cytokine levels were determined by Meso Scale Discovery analysis as per the manufacturer's specifications and are presented here as pg/ml/10^6 cells. Since all mice in the PBS group died after days 5 post-challenge, significance in this study was calculated by comparing groups that were vaccinated with 20 µg DBF + 2.5 µg dmLT and mice vaccinated with BECC438b formulations using Welch t-test. +p<0.05; ++p<0.01; +++p< 0.001.

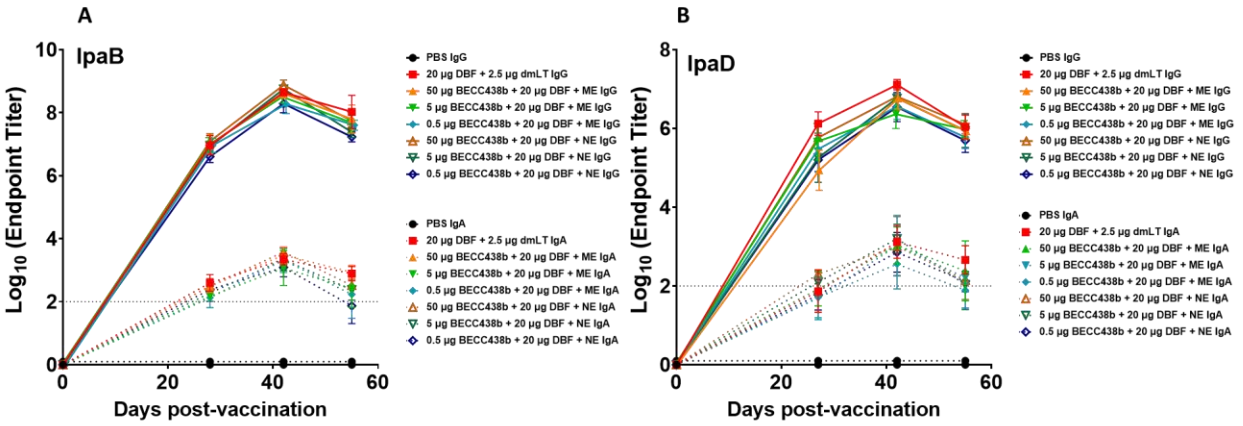

Supplementary Figure S5. Antigen-specific IgG and IgA responses were assessed in mice following IN administration with different formulations. The vaccination groups included PBS, 20 µg DBF + 2.5 µg dmLT, 50 µg, 5 µg, or 0.5 µg BECC438b + 20 µg DBF + ME or NE. Vaccinations were administered three times on Day 0, 14, and 28. Blood and fecal samples were collected, and serum titers for IgG (represented by filled symbols with solid lines) and fecal IgA (represented by open symbols with dashed lines) specific for IpaB (left) or IpaD (right) were determined using ELISA. The individual titers are expressed as EU ml^{-1}. Each data point represents the mean, and the error bars indicate the SD of each group (n=10/group).

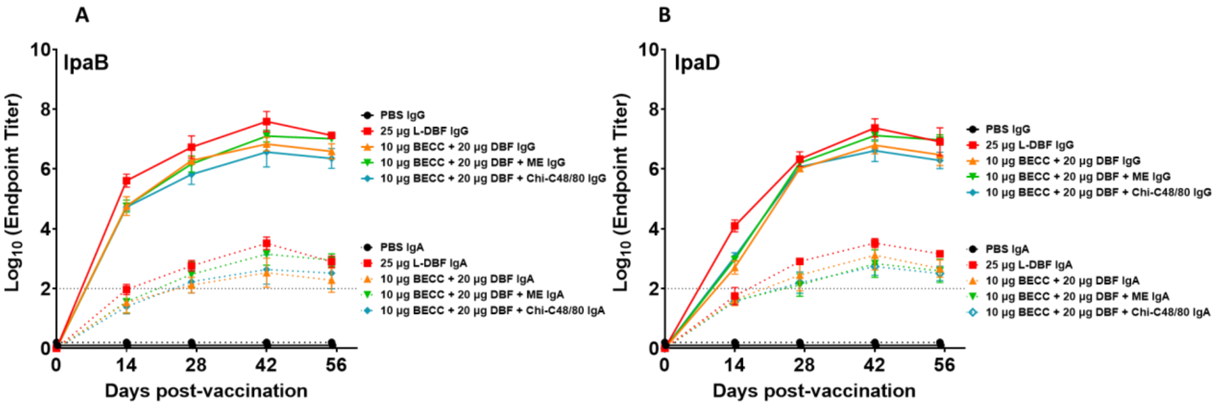

Supplementary Figure S6. Antigen-specific IgG and IgA responses were evaluated in mice following IN vaccination with PBS, 25 µg L-DBF, 10 µg BECC438b + 20 µg DBF with or without ME or with Chi-C48/80 IN three times (Day 0, 14 and 28). Blood and fecal samples were collected, and ELISA was performed to measure serum titers of IgG (represented by filled symbols with solid lines) and fecal IgA (represented by open symbols with dashed lines) specific for IpaB (left) or IpaD (right). The individual titers are represented as EU ml^{-1}. Each point represents the mean and error bars represent SD of each group (n=10/group).

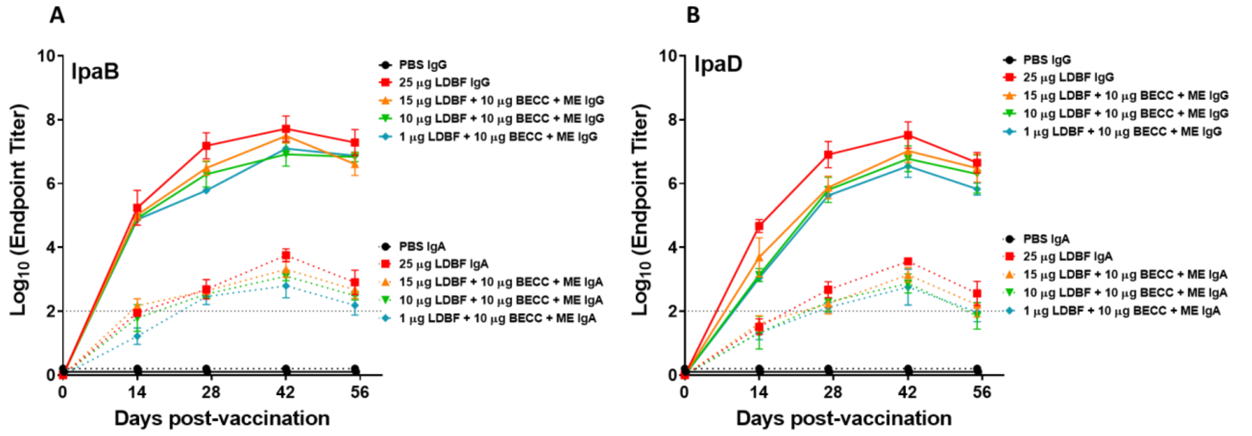

Supplementary Figure S7. Antigen-specific IgG and IgA responses. The experimental groups of mice received IN vaccinations including PBS, 25 µg L-DBF via IN administration, and 10 µg BECC438b combined with 15 µg, 10 µg, or 1 µg L-DBF with ME on three separate occasions (Day 0, 14, and 28). Blood and fecal samples were collected and serum titers for serum IgG (filled symbols with solid lines) and fecal IgA (open symbols with dashed lines) specific for IpaB (left) or IpaD (right) were measured by ELISA. The individual titers are represented as EU ml^{-1}. Each point represents the mean and error bars represent SD of each group (n=10/group).

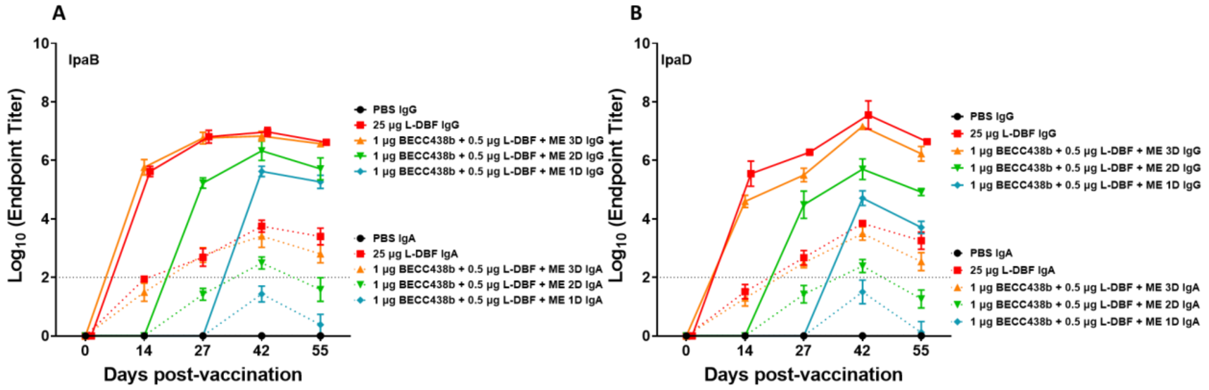

Supplementary Figure S8. Antigen-specific IgG and IgA responses. A separate cohort of mice received either PBS or 25 µg L-DBF intranasally on three occasions (Day 0, 14, and 28). Another cohort of mice received a single prime vaccination of 1 µg BECC438b + 0.5 µg L-DBF + ME via IN route. A second group of mice received a prime vaccination followed by one booster dose (2 doses or 2D), and a third group received a prime vaccination followed by two booster doses (3 doses or 3D). Blood and fecal samples were collected, and serum titers of IgG (represented by filled symbols with solid lines) and fecal IgA (represented by open symbols with dashed lines) specific to IpaB (left) or IpaD (right) were measured using ELISA. The individual titers are expressed as EU ml^{-1}. Each data point represents the mean value, and the error bars represent the standard deviation of each group (n=10/group).
